# Anti-ophidian activity of Bredemeyera floribunda Willd. (Polygalaceae) root extract on the local effects induced by Bothrops jararacussu venom

**DOI:** 10.1590/1414-431X20187581

**Published:** 2018-12-03

**Authors:** N.T.Q. Alves, R.M. Ximenes, R.J.B. Jorge, J.A.M. Silveira, J.V.A. Santos, F.A.P. Rodrigues, P.H.S. Costa, F.A.F. Xavier, J.S.A.M. Evangelista, A. Havt, V.C.G. Soares, M.H. Toyama, A.N.A. Oliveira, R.M. Araújo, R.S. Alves, H.S.A. Monteiro

**Affiliations:** 1 Departamento de Fisiologia e Farmacologia, Universidade Federal do Ceará, Fortaleza, CE, Brasil; 2 Departamento de Antibióticos, Universidade Federal de Pernambuco, Recife, PE, Brasil; 3 Centro Acadêmico de Vitória, Universidade Federal de Pernambuco, Vitória de Santo Antão, PE, Brasil; 4 Faculdade de Veterinária, Universidade Estadual do Ceará, Fortaleza, CE, Brasil; 5 Universidade Paulista, Campus Jundiaí, Jundiaí, SP, Brasil; 6 Instituto de Biociências, Universidade Estadual Paulista, São Vicente, SP, Brasil; 7 Instituto de Química, Universidade Federal do Rio Grande do Norte, Natal, RN, Brasil; 8 Departamento de Análises Clínicas e Toxicológicas, Universidade Federal do Ceará, Fortaleza, CE, Brasil

**Keywords:** Bredemeyera floribunda, Snakeroot, Snake venom, Rutin, Antihemorrhagic, Anti-edematogenic

## Abstract

Bredemeyera floribunda roots are popularly used to treat snakebites in the semiarid region of Northeast Brazil, and previous studies indicate the anti-ophidian actions of triterpenoid saponins found in its roots. To assess B. floribunda root extract (BFRE) activity against the effects of Bothrops jararacussu venom (BjuV), antiphospholipasic, antiproteolytic, antihemorrhagic, antinecrotic, and anti-edematogenic activities were investigated in mice. Phytochemical analysis revealed the presence of saponins, flavonoids, and sugars, with rutin and saccharose being the major constituents of BFRE. Acute toxicity was determined and BFRE was nontoxic to mice. Phospholipase A2 and proteolytic activities induced by BjuV were inhibited *in vitro* by BFRE at all concentrations tested herein. BFRE (150 mg/kg) inhibited paw edema induced by BjuV (50 µg/animal), reducing total edema calculated by area under the curve, but carrageenan-induced paw edema was unchanged. Hemorrhagic and necrotizing actions of BjuV (50 µg/animal) were considerably decreased by BFRE treatment. Thus, BFRE blocked the toxic actions of *B. jararacussu* venom despite having no anti-inflammatory activity, which points to a direct inhibition of venom’s toxins, as demonstrated in the *in vitro* assays. The larger amounts of rutin found in BFRE may play a role in this inhibition, since 3′,4′-OH flavonoids are known inhibitors of phospholipases A2.

## Introduction

Snakebites are a neglected health problem in tropical regions of South America, Africa, Oceania, and Asia (1,2). In Brazil, the main genus of snakes responsible for envenomation is *Bothrops*. Among *Bothrops* species, *B. jararacussu* is responsible for up to 10% of the cases in its area of occurrence, being one of the most feared snakes of Brazil. The effects of envenomation from this genus are characterized by systemic and local effects including, edema, blisters, necrosis, bleeding, and local inflammation (3,4).

The use of plants for treating envenomation caused by snakebites is an age-old practice found in many cultures, long before commercial anti-venoms were developed (5). Plant extracts, which have multiple biochemical constituents, are an extremely rich source of pharmacologically active compounds. Interactions of certain active compounds with toxins and lethal enzymes found in snake venom can lead to neutralization of their toxic activities (6).

The only effective snakebite treatment is the use of mono- or polyvalent antiserum, but it is ineffective for mitigating local damage induced by the venom (7). This has motivated the search for alternative neutralizing agents from a variety of natural sources and development of synthetic compounds aimed at obtaining novel therapeutic tools to complement and improve conventional serum therapy (8,9).

In Brazil, several plant species have been classified as anti-ophidian in ethnobotanical surveys, but only a few have been scientifically evaluated (10,11). Among these species, *Bredemeyera floribunda* Willd. (Polygalaceae), known as "*raiz-de-cobra*" (Portuguese for snakeroot), is widely used to treat snakebites in Brazilian folk medicine. Its anti-ophidian activity is in part attributed to the triterpenoid saponins found in its roots (5,10,12). The aim of this study was to characterize the anti-ophidian activity of an ethanol extract of *B. floribunda* roots on the local effects induced by *B. jararacussu* venom both *in vitro* and *in vivo*.

## Material and Methods

### Plant material, venom, and reagents

Roots of *B. floribunda* Willd. (Polygalaceae) were collected in March 2013 in Serra do Araripe, Ceará, Brazil. Plant identification was performed by taxonomists from Herbarium Prisco Bezerra of Universidade Federal do Ceará, and a voucher was deposited (No. 58399). *B. jararacussu* venom (BjuV) was donated by the Butantan Institute (Brazil). All substrates and reagents used in this work were obtained from Sigma (USA).

### Preparation and phytochemical characterization of *B. floribunda* roots extract (BFRE)

Dried and crushed roots (200 g) were extracted for 7 days with ethanol 70%, three consecutive times. The ethanol was evaporated at 40°C at low pressure and then the extract was lyophilized yielding 14 g of yellow powder. Secondary metabolites in the BFRE were assessed by thin-layer chromatography (TLC) using chemical developers as described in Table S1 (13). Gel chromatography using Sephadex LH-20 (Pharmacia Fine Chemicals, Sweden) eluted with methanol was performed four times yielding five fractions after TLC analysis: A (6.1 g), B (0.5 g), C (0.9 g), D (2.6 g), and E (3.2 g). These fractions were further analyzed by ^1^H and ^13^C nuclear magnetic resonance (NMR).

The 1D and 2D NMR spectra were collected on a Bruker Avance DRX-500 (500 MHz for ^1^H and 125 MHz for ^13^C) spectrometer (USA) equipped with a 5 mm inverse detection z-gradient probe. Chemical shifts (δ) are reported in ppm relative to the residual MeOD (δ 3.31), and to the central peak arising from the MeOD carbon (δ 49.15).

### *In vitro* enzymatic inhibition

*Anti-phospholipase A2 activity*. Phospholipase A2 (PLA2) activity was assessed using 4-nitro-3-(octanoyloxy)benzoic acid (4N3OBA, Biomol, USA) as a chromogenic substrate in 96-well plates. First, BjuV or mixtures of BjuV and BFRE (1:1, 1:2, 1:3, w:w) were diluted in buffer (10 mM Tris-HCl, 10 mM CaCl_2_, 100 mM NaCl, pH 8.0) to a final concentration of 2 mg/mL. Reaction mixtures were prepared using 200 µL of buffer, 20 µL of substrate, 20 µL of extract, and 20 µL of BjuV (9). The absorbance was measured at 425 nm and read every 10 min using a Spectramax 340 plate reader (Molecular Devices^®^, USA).

*Anti-proteolytic activity.* Proteolytic activity was determined using the synthetic chromogenic substrate Nα-benzoyl-DL-arginine p-nitroanilide (DL-BApNA), in 96-well plates, according to Ponce-Soto et al. (14). BjuV or mixtures of BjuV and BFRE (1:1, 1:2, 1:3, w:w) were diluted with buffer (Tris-HCl, 10 mM, 10 mM CaCl_2_, 100 mM NaCl, pH 7.8) at 2 mg/mL. Reaction mixtures were prepared using 200 µL of buffer, 20 µL of substrate, 20 µL of extract, and 20 µL of BjuV. The microplate was subsequently incubated at 37°C and after 30 min, the reaction was stopped by adding 200 µL of 5% trichloroacetic acid and was centrifuged at 2205 *g* for 5 min at room temperature. The microplate was read at 410 nm using a microplate reader VERSA Max plate reader (Molecular Devices^®^).

### Animals

Swiss male mice (30-35 g, n=6 for each group) were used. Animals were housed and kept in a temperature-controlled room (23±2°C) under a 12/12 h light/dark cycle with food and water *ad libitum*. This study was approved by the Ethics Committee on Animal Research of Universidade Federal do Ceará under protocol number 52/13.

### Acute toxicity

A limit test of 2000 mg/kg was performed according to the OECD 425 guideline. First, one animal was administered a dose of 2000 mg/kg followed by 24 h of observation. Since this animal did not die, two more animals received the BFRE (2000 mg/kg, *ip*) under the same conditions. A total of five animals were used, and after a long-term observation of 14 days, the animals were euthanized, and necropsy was performed with histological analysis of their organs (15).

### Experimental groups

Mice were anesthetized with sodium pentobarbital (50 mg/kg, *ip*) and randomized into 6 groups: I: Saline, 50 μL/site; II: BjuV, 50 μg/site; III: BFRE, 150 mg/kg, *ip*, 30 min before BjuV; IV: BFRE, 150 mg/kg, *ip*, 30 min after BjuV; V: BjuV (50 μg) + BFRE (150 μg) (1:3, w/w) pre-incubated for 15 min in total volume of 50 μL; and VI: antibothropic serum (ABS, *ip*) in a sufficient quantity to neutralize 500 mg of venom 30 min before BjuV administration.

For the investigation of carrageenan-induced edema, four additional groups were created: VII: carrageenan group, 1% carrageenan dissolved in 0.9% NaCl solution, 50 μL/paw; VIII: BFRE, 150 mg/kg, *ip*, 30 min before carrageenan; IX: BFRE, 150 mg/kg, *ip*, 30 min after carrageenan; X: indomethacin, 10 mg/kg, *ip*, 30 min before carrageenan.

### Inhibition of paw edema induced by *B. jararacussu* venom or carrageenan

After a sub-plantar injection of 50 μL of BjuV or carrageenan and treatment according to each previously described group (n=6), paw volume was measured at 10, 30, 60, 120, 240, and 480 min using a plethysmometer (Ugo Basile^®^, Italy). The contralateral hind paw was injected with sterile saline (50 μL/paw) in all animals as a volume control. Paw edema was estimated by calculating volume difference compared to basal paw volume (16). For each time-point, values were compared among experimental groups. The total edema was calculated as area under the curve (AUC) of the increase in paw volume.

### Hemorrhage and necrosis inhibition

Mice were anesthetized with sodium pentobarbital (50 mg/kg, *ip*) and dorsal skin was shaved. The venom solution (50 μL) was administered intradermally and treatments were performed according to the previously described experimental groups I to VI (n=12). After 2 h, half of the animals in each group were euthanized, and their dorsal skin was removed. The inner surface of the skin was examined, photographed, and the hemorrhagic area size (mm^2^) was measured using ImageTool 3.00 software (University of Texas Health Science Center, USA) (17). To determine the necrotic area (mm^2^), the remaining animals were euthanized 72 h after their respective treatments, and the same procedure was performed.

### Histopathological analysis

Tissues samples from paw edema experiments were collected immediately after euthanizing the animals and stored in 10% buffered formalin for 24 h, followed by 70% ethanol. Fragments were subjected to dehydration, diaphanization, and cut to a thickness of 4 μm. Slides were stained with hematoxylin-eosin (HE) and analyzed using a trinocular optical microscope Motic^®^ BA310 (China) with an attached camera (2.0 MP Live Motic^®^ 2000 resolution), using the Motic^®^ Image Plus 2.0 software.

### Statistical analysis

All results are reported as means±SE. The normal distribution was analyzed by D’Agostino-Pearson test and the data were analyzed by ANOVA followed by Bonferroni post-test when several experimental groups were compared to the control group. Results were considered significant when P<0.05.

## Results

### Phytochemical characterization of *B. floribunda* roots extract

TLC analysis of BFRE revealed the presence of flavonoids, saponins, and sugars. Sephadex LH-20 gel chromatography provided five fractions (A to E) containing chemically distinct groups of compounds. Subsequent ^1^H and ^13^C NMR analysis identified fraction A as a mixture of triterpene saponins and saccharose, fraction B as saccharose, and fraction C as a mixture of rutin and saccharose. After rechromatography, fraction D and E yielded 5.3 g of a pure orange powder soluble in methanol (B1, 37.8% of the extract).

The ^1^H NMR spectrum (MeOD, 500 MHz, Figure S1) of compound B1 showed characteristic glycoside hydrogen signals between δ 3.26 and 3.81 ppm. The chemical shifts characteristic of aromatic hydrogens between δ 6.19 and 7.67 ppm were also observed. Upon analyzing the region between δ 6.19 and 7.67 (Figure S2), it was possible to identify the peaks at δ 6.19 (H_6_, d, *J*=1, 8 Hz) and δ 6.38, but it was not possible to calculate *J* due to lack of multiplicity. However, the *J* values for H_6_ and ^13^C NMR signals indicated meta coupling between them. Signals for H_5′_ and H_6′_ located in ring B, at δ 6.87 (H_5′_, d, *J*=6.7 Hz) and δ 7.62 (H_6′_, dd, *J*=1.4 Hz e 6.7 Hz) ppm, with ortho and meta coupling were observed. In ring B, a signal at δ 7.67 (H_2′_, d, *J*=1.36 Hz) arising from meta coupling with H_6'_ was also found.

The ^13^C NMR spectrum (100 MHz, MeOD, Figure S3) exhibited a signal at δ 18.0 ppm, characteristic of methyl carbon. Between δ 68.6 and 104.8 ppm, signals from oxygenated carbons with sp^3^ hybridization were observed, which are characteristic of glycosides, along with signals originated from anomeric carbons at δ 102.5 (C-1-′) and 104.8 (C-1-). Between δ 105.7 and 166.0 ppm, signals arising from sp^2^ carbons belonging to aromatic rings were observed. An α,β-unsaturated ketone carbonyl signal at δ 179.4 ppm was also seen in NMR spectrum.

Heteronuclear single quantum correlation (HSQC) analysis (C_5_D_5_N_,_ 500 MHz, Figure S4) corroborated the proposed hydrogenation profile for each carbon (Table 1). The NMR spectra, in the context of literature data, supported the identification of compound B1 as the flavonoid rutin (18).

**Table 1:** ^1^H nuclear magnetic resonance (NMR) (MeOD, 400 MHz) and ^13^C NMR (MeOD, 100 MHz) data of compound B1.

C	NMR ^13^C	NMR ^13^C (Moura et al., 2011)	NMR ^1^H (int., mult., *J*_H,H_)	NMR ^1^H (Moura et al., 2011) (int., mult., *J*_H,H_)
2	159.4	156.5		
3	145.9	133.2		
4	179.4	177.3		
5	163.0	161.2		
6	100.0	98.7	6.19 (1 H, d, J = 1.4 Hz)	6.20 (1 H, d, J = 2.1 Hz)
7	166.0	164.5		
8	95.0	93.6	6.38 (1 H, d, J = 1.4 Hz)	6.40 (1 H, d, J = 2.1 Hz)
9	158.5	156.4		
10	105.7	103.7		
1′	135.7	121.5		
2′	123.4	116.2	7.67 (1 H, d, 1.4 Hz)	7.53 (1 H, d, J = 2.0 Hz)
3′	117.8	115.2		
4′	149.8	148.5		
5′	116.1	115.2	6.87 (1 H, d, J = 6.7 Hz)	6.83 (1 H, d, J = 9.0 Hz)
6′	123.2	121.0	7.62 (1 H, dd, J = 1.4 Hz and J = 6.7 Hz)	7.75 (1 H, dd, J = 2.4 Hz and J = 7.8 Hz)
1″	104.8	101.2		
2″	77.2	75.8		
3″	75.8	74.0		
4″	71.4	69.9		
5″	78.2	76.4		
6″	68.6	66.9		
1‴	102.5	100.7		
2‴	72.3	70.5		
3‴	72.1	70.3		
4‴	74.0	71.8		
5‴	68.9	68.2		
6‴	18.0	17.6		

### Acute toxicity test

No deaths occurred up to a 2000 mg/kg (*ip*) BFRE dosage, but the animals showed mild drowsiness in the first 6 h after administration. Neither necropsy nor histopathological analysis indicated any changes in the organs. The LD_50_ of BFRE is greater than 2000 mg/kg, indicating that BFRE has low acute toxicity (data not shown).

### *In vitro* enzymatic inhibition

**Figure 1:**
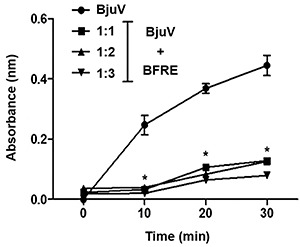
Effect of *Bredemeyera floribunda* root extract (BFRE) on the phospholipase A2 activity of *Bothrops jararacussu* venom (BjuV). BFRE was preincubated with BjuV at ratios of 1:1, 1:2, and 1:3 (BjuV:BFRE). 4N3OBA was used as a substrate and increasing absorbance was measured at 425 nm. Data are reported as means±SE. *P<0.05 compared to BjuV (ANOVA with Bonferroni post-test).

**Figure 2:**
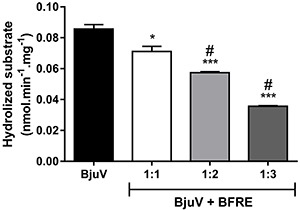
Effect of *Bredemeyera floribunda* root extract (BFRE) on the proteolytic activity of *Bothrops jararacussu* venom (BjuV). BFRE was preincubated with BjuV at ratios of 1:1, 1:2, and 1:3 (BjuV:BFRE). DL-BApNA was used as a substrate and increasing absorbance was measured at 410 nm. Data are reported as means±SE. *P<0.05, ***P<0.001 compared to BjuV; ^#^P<0.05 compared among BFRE 1:1 ratio (ANOVA with Bonferroni post-test).

PLA2 and proteolytic activities of BjuV were inhibited *in vitro* by BFRE at different ratios (1:1, 1:2, and 1:3, w/w) as shown in Figures 1 and 2, respectively. Proteolytic activity was inhibited in a concentration-dependent manner, while PLA2 activity did not show a concentration-dependent inhibitory relationship.

### Anti-edematogenic activity

**Figure 3:**
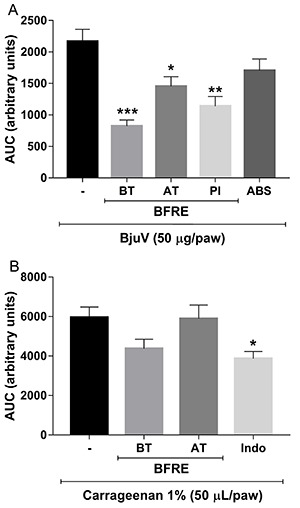
Effect of *Bredemeyera floribunda* root extract (BFRE) on *Bothrops jararacussu* venom (BjuV)-induced (*A*), and carrageenan-induced (*B*) paw edema. Paw volume was measured at 10, 30, 60, 120, 240, and 480 min after BjuV inoculation and total edema was calculated as AUC (area under curve). Data are reported as means±SE. *P<0.05, **P<0.01, and ***P<0.001 compared to BjuV/carrageenan (control) (ANOVA with a Bonferroni post-test). BT: group treated with BFRE (150 mg/kg, ip) 30 min before BjuV/carrageenan inoculation; AT: group treated with BFRE (150 mg/kg, *ip*) 30 min after BjuV/carrageenan inoculation; PI: BjuV and BFRE (1:3) preincubated for 15 min at 37°C before inoculation; ABS: antibothropic serum; Indo: indomethacin (10 mg/kg, *ip*) 30 min before carrageenan inoculation.

Intraperitoneal treatment with BRFE (150 mg/kg) either before or after BjuV administration reduced venom-induced increases in paw volume (Figure 3A). When BjuV was mixed with BFRE (1:3) before injection into the mouse paw, edema was also inhibited. Edema inhibition by BFRE administered before BjuV injection was long lasting compared to the other experimental conditions.

To characterize a possible anti-inflammatory effect of BFRE, another experimental group of animals was treated with 1% carrageenan. BFRE administered 30 min before carrageenan inhibited the increase in paw volume seen at 240 min, as also observed in the indomethacin group. However, total edema as determined by AUC was only inhibited in the indomethacin group (Figure 3B).

**Figure 4:**
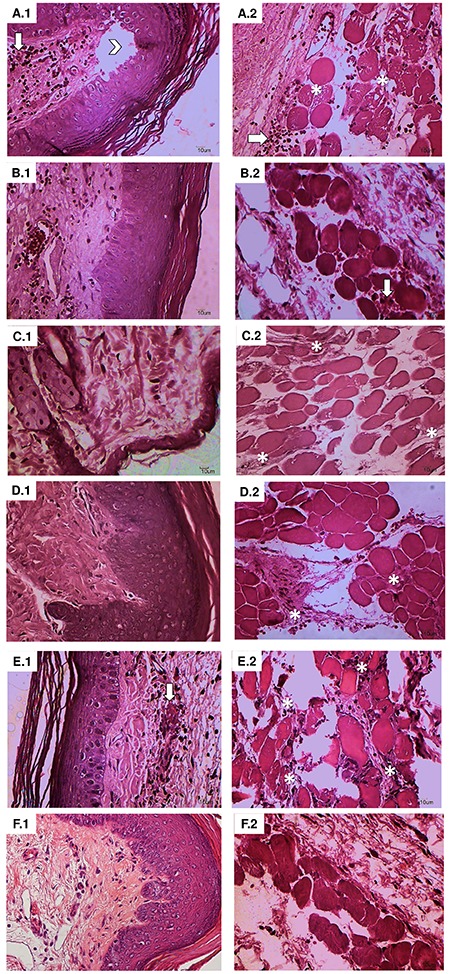
Histological analysis of edema induced by *Bredemeyera jararacussu* venom and neutralization by *Bothrops floribunda* extract. Epidermis/dermis (*1*) and Hypoderm/muscle tissue (*2*). *A.1/A.2:* Sub-plantar injection of BjuV 50 µg/50 uL; *B.1/B.2:* Pre-treatment *ip* with BFRE 150 mg/kg; *C.1/C.2:* Post-treatment *ip* with BFRE 150 mg/kg; *D.1/D.2:* Pre-incubated BFRE:BjuV, 1:3 w/w; *E1/E.2:* antivenom serum, sufficient quantity to neutralize 500 g of the venom; *F.1/F.2:* saline control. *A:* Edema (arrow head). *A* to *E:* Edema, inflammatory cells, and destruction of muscle fibers at different levels (asterisks). *A, B,* and *E:* mild hemorrhagic areas (arrows). *F:* no alterations. Light microscope at ×400, HE staining. Scale bar: 10 µm.

Histopathological analysis of paw tissue indicated edema, inflammatory infiltrate, necrosis, and degeneration in muscle fibers in the BjuV group. BFRE treatment before or after BjuV inoculation decreased the number of inflammatory cells and mitigated muscle degeneration. When BjuV and BFRE were mixed in a 1:3 ratio before inoculation, histological analysis indicated the presence of inflammatory cells and muscle degeneration. In the ABS group, edema, inflammatory cells infiltrating the dermis and subcutaneous tissue, and muscle degeneration were observed (Figure 4).

### Hemorrhagic and necrotic activity

**Figure 5:**
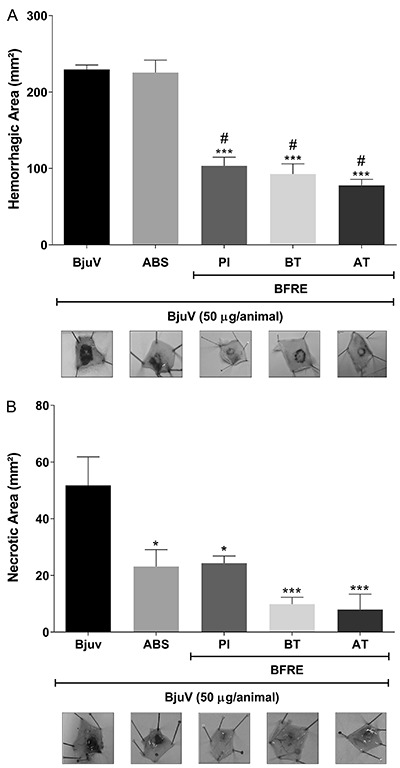
Effect of *Bredemeyera floribunda* root extract (BFRE) on hemorrhagic (*A*) and necrotizing (*B*) effects of *Bothrops jararacussu* venom (BjuV). Lesion areas were calculated in mm^2^. Data are reported as means±SE. *P<0.05, ***P<0.001 compared to BjuV; ^#^P<0.05 compared to ABS (ANOVA with Bonferroni post-test). BT: group treated with BFRE (150 mg/kg, *ip*) 30 min before BjuV inoculation; AT: group treated with BFRE (150 mg/kg, *ip*) 30 min after BjuV inoculation; PI: BjuV and BFRE (1:3) preincubated for 15 min at 37°C before inoculation; ABS: antibothropic serum.

Figure 5A shows a decrease in the hemorrhagic area induced by BjuV due to treatment with BFRE in all experimental groups (III, IV and V). ABS treatment (VI) was not able to inhibit hemorrhaging induced by BjuV. In terms of necrotizing activity, BFRE blocked the venom action in the treated groups, even after 72 h of administration. At this time, a positive control performed with the ABS decreased necrotic areas (Figure 5B), indicating that ABS required some time to reach an effective concentration in tissues.

## Discussion

The anti-ophidian properties of natural products have been exhaustively studied. Ubiquitous secondary metabolites possess anti-ophidian activity and include flavonoids, steroids, and triterpenes (10). Bredemeyerosides B and D, triterpene saponins isolated from the roots of *B. floribunda* Willd., exhibited significant anti-ophidian activity in mice, although the therapeutic activity of the associated extract remains poorly characterized (12,19). Flavonoids and other phenolics, such as rutin, quercetin, and chlorogenic acid, have been suggested to be responsible for the anti-ophidian activity of several plants used in traditional medicines to treat snakebites, such as *Casearia sylvestris* Sw. (Flacourtiaceae), *Vellozia flavicans* Mart. ex Schult. (Velloziaceae), and *Vochysia haenkeana* Mart. (Vochysiaceae) (4,20,21).

The local toxic effects caused by bites of snakes from the Viperidae family, especially those from the Crotalinae subfamily, are mainly due to the actions of PLA2, metalloproteases, and serine proteases. Snake venom metalloproteases and PLA2 are poor immunogenic and fast-acting toxins, making complete inhibition of local effects in a clinical setting very difficult (22). Snake venom PLA2 can be catalytically active (Asp49) or inactive (Lys49), but both are myotoxic and can trigger inflammatory responses causing edema, which is mediated to different degrees by amines (histamine and serotonin) released by degranulating mast cells (23). The *in vitro* PLA2 activity of BjuV on 4N3OBA was significantly inhibited by BFRE. Flavonoids with 3′- and 4′-OH groups (such as quercetin and rutin) are well-known inhibitors of snake venom PLA2 enzymatic and toxic activities (24,25,26).

BFRE also inhibited the paw edema induced by BjuV. Wanderley et al. (27) characterized the local inflammation caused by *B. jararacussu* and inferred that the venom produces an early edema onset dependent on prostanoid production and neutrophil migration. These effects were suggested to be mediated by increased local production of IL-1β, COX-2 expression, and neutrophil chemotaxis induced by the venom. To determine if the effect of BFRE on paw edema was due to a general anti-inflammatory action or direct inhibition of BjuV toxins, the effect of BFRE on carrageenan-induced paw edema was evaluated. Paw edema induced by carrageenan is a widely used model to investigate the pathophysiology of acute inflammation and to assess anti-inflammatory effects of anti-inflammatory drug candidates (28). Intraplantar injection of carrageenan produced acute inflammation characterized by increased water in the tissue, exudation of plasma proteins, infiltration of neutrophils, and arachidonic acid metabolism through the cyclooxygenase and lipoxygenase pathways. The maximum effects of carrageenan administration can be observed after approximately 3 h and involve the synthesis and release of mediators such as prostaglandins, histamine, bradykinin, serotonin, and leukotrienes (29).

BFRE treatment decreased the edematogenic peak effects at 240 min but did not inhibit the total edema reported as the AUC. Rutin has been shown to exert anti-inflammatory effects in rat paw edema induced by carrageenan through the inhibition of neutrophil chemotaxis (30). In addition to enzymatic inhibition of venom toxins, BFRE may decrease neutrophil migration to the site as well as the production of inflammatory mediators, decreasing the edema severity in carrageenan- and BjuV-induced paw edema.

The other major group of toxins responsible for the local effects in bothropic envenomation are snake venom zinc-dependent metalloproteases (SVMPs). To evaluate the inhibitory effects of BFRE on SVMP, *in vitro* inhibition using DL-BApNA as a substrate was monitored. BFRE inhibited BjuV proteolytic activity in a concentration-dependent manner. SVMPs are the main toxins involved in hemorrhaging and necrosis caused by bothropic envenomation. The pathogenesis of venom-induced bleeding involves direct damage to microvessels, which is related to the proteolytic activity of SVMPs (31). SVMPs are mainly responsible for the hemorrhaging through the degradation of the endothelial layer of basement membrane proteins such as laminin, fibronectin, type IV collagen, and proteoglycans. Furthermore, SVMPs may also inhibit platelet aggregation and trigger cytokine release (31,32).

In this study, BFRE was shown to inhibit both the hemorrhagic and necrotizing effects of BjuV. It is likely that BFRE chelated the zinc of the metalloproteinases, or inhibit degradation of the components of the basement membrane, preventing the action of these enzymes and mitigating the hemorrhagic, proteolytic, and necrotizing effects of BjuV. Flavonoids isolated from *Schizolobium parahyba* have been shown to inhibit the proteolytic, hemorrhagic, and necrotizing activities of *B. jararacussu* and *B. neuwiedi* venoms (33).

Previous studies indicated that the efficacy of extracts is greater than that of single constituents, likely due to the synergism among molecules present in the extract. Extracts rich in proanthocyanidins, triterpenoid saponins, polysaccharides, and coumarins are potent inhibitors of SVMPs found in *Bothrops* venom (22). On the other hand, the inhibition of snake venom PLA2 by natural compounds is often due to the association of PLA2 and flavonoids present in these plants. BFRE contains both flavonoids and saponins, which may contribute in a synergistic manner to the observed effects (34).

In conclusion, BFRE inhibited *B. jararacussu* whole venom PLA2 and protease enzymatic activities *in vitro*, and their related toxic effects *in vivo* (edema and hemorrhage/necrosis, respectively). Previous reports indicated the anti-ophidian action of triterpenoid saponins found in *B. floribunda* roots (19). The large amounts of rutin found in *B. floribunda* roots could potentiate the effect of saponins, since 3′,4′-OH flavonoids are well-known inhibitors of PLA2. These results provide some evidence for the use of *B. floribunda* root extract in Brazilian traditional medicine. More studies are required to elucidate the mode of action of *B. floribunda* and to consider its use as a possible adjuvant in treatment of local effects of bothropic envenomation.

## Supplementary material

Click here to view [pdf].
